# Switching porosity of stable triptycene-based cage *via* solution-state assembly processes†

**DOI:** 10.1039/d0ra00128g

**Published:** 2020-03-03

**Authors:** Hui Ma, Tian-Long Zhai, Zhen Wang, Guang Cheng, Bien Tan, Chun Zhang

**Affiliations:** College of Life Science and Technology, National Engineering Research Center for Nanomedicine, Huazhong University of Science and Technology Wuhan 430074 China chunzhang@hust.edu.cn; School of Chemistry and Chemical Engineering, Huazhong University of Science and Technology Wuhan 430074 China

## Abstract

It is a great challenge to tune the porosity of porous materials. As most porous organic cages are soluble, solution processability can be a possible way to regulate the porosity of such materials. Herein, a triptycene-based cage (TC) is demonstrated to be stable in acid, base or boiling water. Meanwhile, its porosity can be tuned by adjusting the solution-state assembly processes. TC molecules crystallized slowly from solution exhibit nearly no porosity to nitrogen (off-state). While, after rapid precipitating from methanol/dichloromethane solution, the obtained TC (TC-rp) is in a porous state and exhibit a high BET surface area of 653 m^2^ g^−1^ (on-state).

## Abstract 2

Here, a kind of triptycene-based cage is demonstrated to have good chemical stability in acid, base and boiling water. Moreover, its porosity can be tuned by varying the solution-state assembly processes.

## Graphical abstract

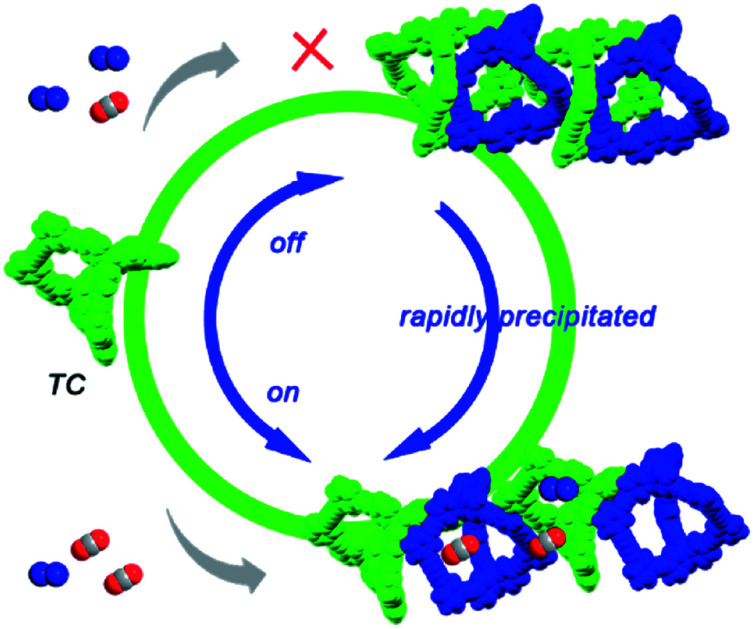

## Introduction

Porous organic cages,^1^ one of the most important subclasses of porous molecular materials,^2–5^ have been recognized as an attractive functional material which could be complementary to established porous network polymers and frameworks (such as metal–organic frameworks (MOFs),^6^ covalent organic frameworks (COFs)^7,8^ or porous organic polymers (POPs)^9^) because of their distinct features like high porosity, good chemical stability, and solution processability. Different from network polymers and frameworks, organic cages contain “extrinsic” and “intrinsic” pores, which refer to the pores located between molecules or within molecules, respectively.^10^ In principle, the organic cages are porous to some guests when their intrinsic and extrinsic pores are interconnected, which is deeply influenced by the assembly patterns.^1^ Such characteristic offers an opportunity to control the porosity of organic cages which is not available for insoluble organic and inorganic frameworks.

Versatile POCs have been successfully synthesized by diverse building blocks and synthetic methods and applied in different fields with varying porosity.^11–15^ However, most POCs exhibit non-porosity to the guest molecules because the internal cavities of the cages are blocked by their window-to-arene assembly patterns.^16,17^ To tune the POCs' porosity from off to on, coupling POCs into frameworks could be a fashionable method, while such cage-based polymeric frameworks were generally constructed by covalent synthesis or coordination chemistry, which would lead to the sacrifice of their solution processability.^18–22^ Other methods were also developed, for example, Cooper and co-workers realized guest-induced “on–off” porosity transformation by virtue of POCs' noncovalent intermolecular packing.^23,24^ Doonan *et al.* controlled the porosity of POC by kinetic methods.^25^ In a recent study of Banerjee *et al.*, non-porous organic cage could be converted to a porous polymorph by treatment with DMF.^26^ But still, the porosity tuning is still a challenge for POCs.

Triptycene, with rigid three-dimensional structure, is an attractive building block for porous materials^27–30^ and supramolecular hosts.^31–34^ Mastalerz's group synthesized a series of remarkable triptycene-based cages through reversible reactions of imine chemistry or boronic acid condensation.^35–39^ The cages based on reversible reactions were always obtained with high yields because of the self-correcting mechanism. Meanwhile, they usually lacked good chemical stability. Since organic cages with better chemical stability have more widespread applications, recently, Mastalerz's group also obtained several chemically stable organic cages based on previously reported imine cages by the conversion of imine groups to more stable amide groups or carbamate groups.^40,41^ But such stable triptycene-based cages were rarely reported. Herein, we found the ethynylene-linking triptycene-based cage (TC) from a Glaser coupling reaction had excellent stability in acid, base or boiling water. Moreover, the porosity of TCs were tunable by controlling their assembly processes.

## Results and discussion

**Scheme 1:**
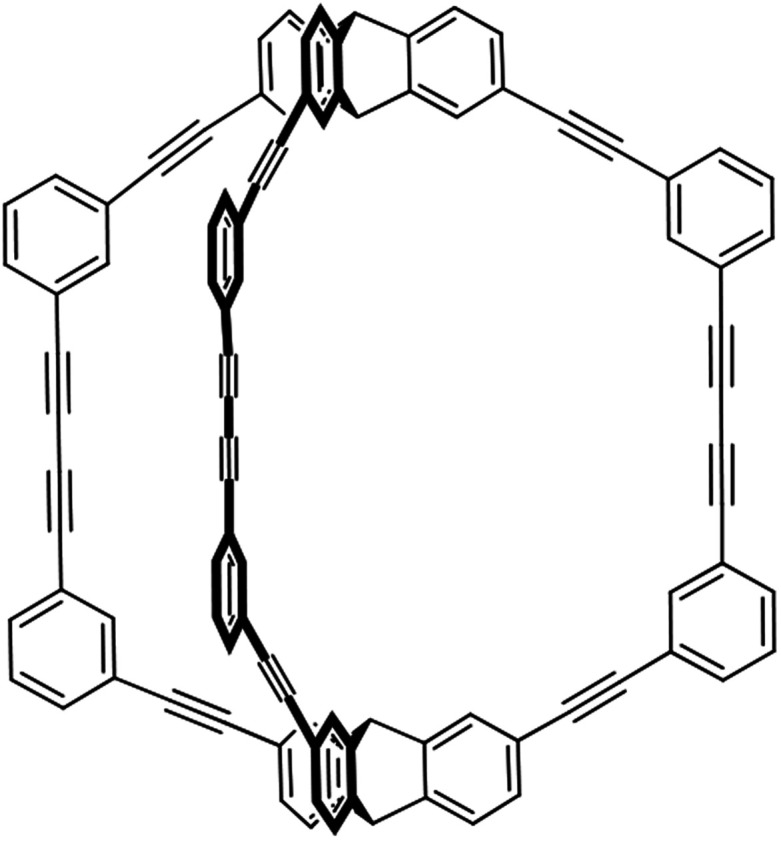
Chemical structure of triptycene-based cage (TC).

The triptycene-based cage (TC) (Scheme 1) was synthesized by the copper-mediated modified Eglinton–Glaser oxidative coupling reaction.^42^ As shown in the spectrum of Proton nuclear magnetic resonance (^1^H NMR) (Fig. S1†), there were signals at *δ* = 5.50 and 5.57 ppm for the inner and outer bridgehead protons and signals appeared from *δ* = 7.32 to *δ* = 7.77 for all aromatic protons, respectively. The Fourier-transform infrared (FT-IR) spectrum of TC showed a peak at 2214 cm^−1^ corresponding to the acetylene signal (Fig. S2†), which was similar with the previous literature.^42^ The single crystal of TC was obtained by slow evaporation of a CH_2_Cl_2_/1,3,5-trimethylbenzene solution. With space group of *P*1̄, each cell (*a* = 18.4087(7) Å, *b* = 20.1018(8) Å, *c* = 21.0854(8) Å, *α* = 113.054(4)°, *β* = 97.737(3)°, *γ* = 114.431(4)°) contained two cage molecules and twelve 1,3,5-trimethylbenzene molecules. The solvent molecules were distributed disorderly around cage molecules.

**Fig. 1:**
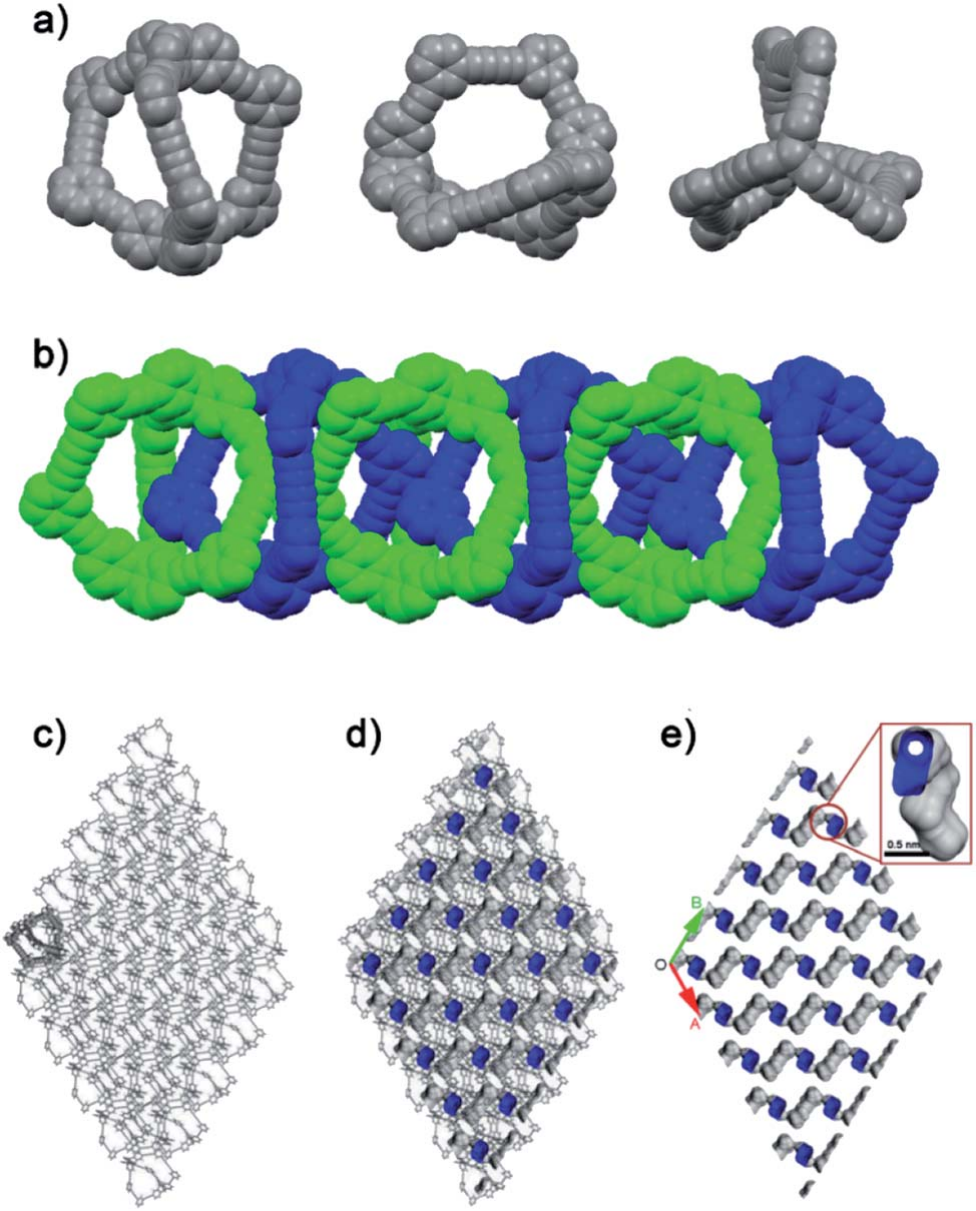
(a) X-ray crystal structures of TC. (b) Six adjacent molecules of TC packing in a window-to-arene fashion (two adjacent TC molecules were presented by different colors). (c) 3D-stacking mode of crystal TCs (hydrogen atoms and solvent molecules are omitted for clarity). And the cross-sectional images of the packing structures with (d) and without (e) TC framework showed that TC has nonconnective lattice voids, as illustrated by the blue Connolly surface (probe radius = 1.82 Å) applied to the crystal structure for the desolvated material.

**Fig. 2:**
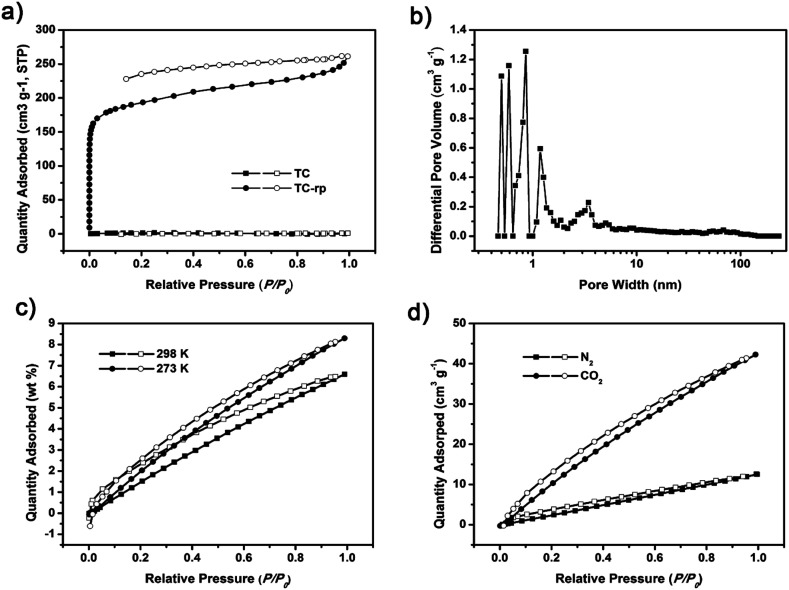
(a) Nitrogen sorption and desorption isotherms of TC and TC-rp at 77 K. (b) Pore size distribution calculated of TC-rp. (c) CO_2_ adsorption and desorption isotherms of TC-rp at 273 K and 298 K. (d) CO_2_ and nitrogen sorption and desorption isotherms of TC-rp at 273 K.

As shown in Fig. 1a, TC exhibited a helical chiral feature with twisted acetylenic units of 176.04–178.75°. The length of the cages was about 18.213 Å. In the crystal cell, although the phenylene units were not parallel with adjacent benzene rings of triptycene moieties, the size of dihedral angles could be only 8.32°, and the vertical dimension between the centroid of benzene ring and the phenylene unit was about 3.5 Å, which suggested the existing of π–π interactions^43–45^ (Fig. S3†). By virtue of C–H⋯π interactions, an interlaced supramolecular structure of TC in the solid state was formed.^42^ Specifically, TC molecules packed in a window-to-arene fashion where the internal cavity of one cage was occupied by phenylene units of the neighboring cage molecules (Fig. 1b). As a consequence, the “intrinsic” pores were blocked, while the “extrinsic” pores could not connect to each other. The simulated accessible pore space of N_2_ (Fig. 1c–e) showed TC had many discrete pores, but these pores were connected by one-dimensional channels with diameter of only 0.2 nm, which would make TC non-porosity to N_2_, given that the kinetic radius of N_2_ is 1.82 Å. In line with the simulation results, nitrogen sorption isotherms of TC were measured at 77 K to evaluate its surface area and porous properties. As shown in Fig. 2a, TC could hardly absorb N_2_ at 77 K with the Brunauer–Emmett–Teller (BET) surface area of only 7 m^2^ g^−1^ (Langmuir surface area was 12 m^2^ g^−1^) (Fig. S4†).

After dissolving TC in dichloromethane, TC-rp was obtained by subsequently addition of methanol. The ^1^H NMR and FT-IR spectra of TC-rp were almost the same as that of TC (Fig. S5 and S6†), demonstrating that the cage's molecular structure did not change during rapid precipitation. However, powder X-ray diffraction (PXRD) study exhibited that the aggregate structure of the cages changed in the process (Fig. S7†). To be specific, two peaks at 12.2° and 17.4° missed in the XRD pattern of TC-rp compared to that of TC, and the other peaks became broader as well, suggesting that the microcrystalline powder TC-rp could stack in a more disordered mode. A plausible reason could be that rapid precipitation of TC-rp from solvent could not afford the whole crystallization process of the cage.

Nitrogen sorption isotherms of TC-rp were also measured at 77 K (Fig. 2a). Contrast to TC, the BET surface area of TC-rp was calculated to be 653 m^2^ g^−1^ (Langmuir surface area was 876 m^2^ g^−1^) (Fig. S8†). It seemed that disordered structure could result in high surface area, which could be caused by the reason that the window-to-arene packing fashion of TC was broken in TC-rp, and then the discrete voids in the cages could connect to each other. TC-rp exhibited typical type I reversible sorption profile, where steep nitrogen uptake could be found at low relative pressure (*P*/*P*_0_ < 0.001), suggesting the existence of abundant micropores. The pore size distribution calculated using DFT method also confirmed the presence of micropore structure (Fig. 2b). The CO_2_ sorption properties of TC-rp were measured at 273 and 298 K, and it could uptake 8.3 wt% CO_2_ at 273 K and 6.6 wt% at 298 K (Fig. 2c), respectively, which could be comparable with other materials of its type. Using the slopes at low pressure in the Henry's law region for both CO_2_ and N_2_ at 273 K, the CO_2_/N_2_ selectivity of 4.8 was calculated for TC-rp (Fig. 2d and S9†).

**Fig. 3:**
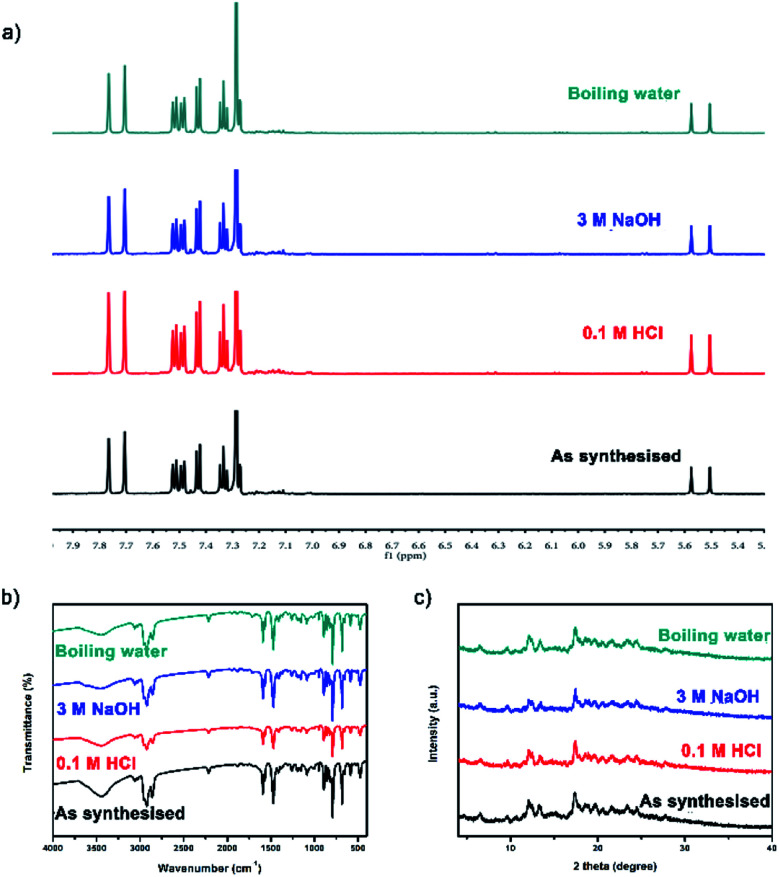
Before and after treatment with acids, bases or boiling water: (a) ^1^H NMR spectra; (b) FT-IR spectra; (c) PXRD patterns.

TC was synthesized by Eglinton–Glaser oxidative coupling reaction, and the covalent bonds in the cages mainly consisted of C–C bonds, which provided chemical stability to the cages. After treatment with 0.1 M HCl, 3 M NaOH and boiling water for more than five days, the ^1^H NMR spectra, the FT-IR spectra and the PXRD patterns of TC before and after treatment were basically the same (Fig. 3), confirming that the structure of TC maintained throughout the whole process, which could be a conclusive evidence for the chemical stability of TC.

## Conclusions

In summary, a triptycene-based cage (TC) synthesized by the copper-mediated modified Eglinton–Glaser oxidative coupling reaction was demonstrated to have excellent chemical stability in acid, base or boiling water. The single crystal structure of TC showed a window-to-arene fashion which blocked its porosity, after rapid precipitation in a methanol/dichloromethane solution, TC-rp was porous to N_2_ and CO_2_ with high BET surface area and good CO_2_ uptake capacity. The development of more smart porous organic cages are undergoing in our lab.

## Methods

### Synthesis of triptycene-based cage (TC)

TC was synthesized following the literature:^42^ the mixture of CuCl (223 mg, 2.25 mmol), Cu(OAc)_2_ (546 mg, 3.0 mmol) and dry pyridine (15 mL) was stirred under argon at 60 °C for 20 min. Then a solution of 2,7,14-tri[(4-ethynylphenyl)ethynyl]triptycene (30 mg, 0.05 mmol) in dry pyridine (3 mL) was added dropwise, and the mixture was stirred at the same temperature for another 10 h. After cooling down to room temperature, the solvents were removed under reduced pressure, and the residue was dissolved in CH_2_Cl_2_ (50 mL) and washed with 1 M aqueous HCl (3 × 30 mL). The aqueous solution was extracted with CH_2_Cl_2_ (2 × 30 mL), the organic phase was combined and dried with Na_2_SO_4_. TC was purified by chromatography with silica gel (CH_2_Cl_2_/petroleum ether, 1 : 4, v/v) to give a white solid (12 mg, 39.6%).

Crystallographic data for TC (C_154_H_118_): *M*_r_ = 1968.64, triclinic, space group *P*1̄, *a* = 18.4087(7) Å, *b* = 20.1018(8) Å, *c* = 21.0854(8) Å, *α* = 113.054(4)°, *β* = 97.737(3)°, *γ* = 114.431(4)°, *V* = 6113.3(5) Å3, *Z* = 2, *ρ*_calcd._ = 1.047 g cm^−3^, *μ* = 0.447 mm^−1^, reflections collected 40 251, data/restraints/parameters 23 863/146/1345, GOF on *F*^2^ 1.317, final *R*_1_ = 0.1355, *wR*_2_ = 0.3514, *R* indices (all data): *R*_1_ = 0.1755, *wR*_2_ = 0.3947, largest diff. peak and hole: 1.51 and −0.76 e Å^−3^. Which is similar to the literature:^42^ C_136_H_94_: *M*_r_ = 1728.11, triclinic, space group *P*1̄, *a* = 18.417(3) Å, *b* = 20.223(3) Å, *c* = 21.134(3) Å, *α* = 101.472(4)°, *β* = 109.674(2)°, *γ* = 114.6330(10)°, *V* = 6185.7(17) Å^3^.

## Conflicts of interest

There are no conflicts to declare.

## Supplementary Material

RA-010-D0RA00128G-s001
