# Supplementary material for: Switching porosity of stable triptycene-based cage via solution-state assembly processes
Source: RSC Adv. 2020 Mar 3;10(15):9088–92. doi: 10.1039/d0ra00128g (PMC9050043; doi:10.1039/d0ra00128g)
Supplement: RA-010-D0RA00128G-s001 [file RA-010-D0RA00128G-s001.pdf]

Electronic Supplementary Material (ESI) for RSC Advances.
This journal is © The Royal Society of Chemistry 2020

Electronic Supplementary Information

Switching Porosity of Stable Triptycene-Based Cage via Solution-State Assembly Processes

Hui Ma,[a,#] Tian-Long Zhai,[a,#] Wang Zhen,[a,#] Guang Cheng,[b] Bien Tan,[b] Chun Zhang.[a,*]

[†]College of Life Science and Technology, National Engineering Research Center for Nanomedicine, Huazhong University of Science and Technology, Wuhan, 430074, China

[‡]School of Chemistry and Chemical Engineering, Huazhong University of Science and Technology, Wuhan, 430074, China

Materials and methods

Preparation of Compounds. TC was synthesized through the literature[S1], and the other chemicals were obtained from commercial sources without further purification.

Methods. ^{1}H nuclear magnetic resonance (NMR) spectra were recorded on Bruker model AV400 NMR spectrometers, where chemical shifts were determined with TMS. Fourier transform infrared (FT-IR) spectra were recorded on a Bruker model VERTEX 70 infrared spectrometer. X-ray diffraction data were recorded on a PANalytical B.V. model X'Pert PRO diffractometer by depositing powder on glass substrate, from $2\theta = 3°$ up to 60° with 0.02° increment.

Gas Sorption Analysis. Surface areas and pore size distributions were measured by nitrogen adsorption and desorption at 77 K using a Micromeritics ASAP 2020 volumetric adsorption analyzer. Sample was degassed at 120 °C for 8 h under vacuum before analysis. CO_2 isotherms were measured at 273 and 298 K up to 1.0 bar using a Micromeritics ASAP 2020 volumetric adsorption analyzer with the same degassing procedure.

Procedures for Preparation of Starting Materials

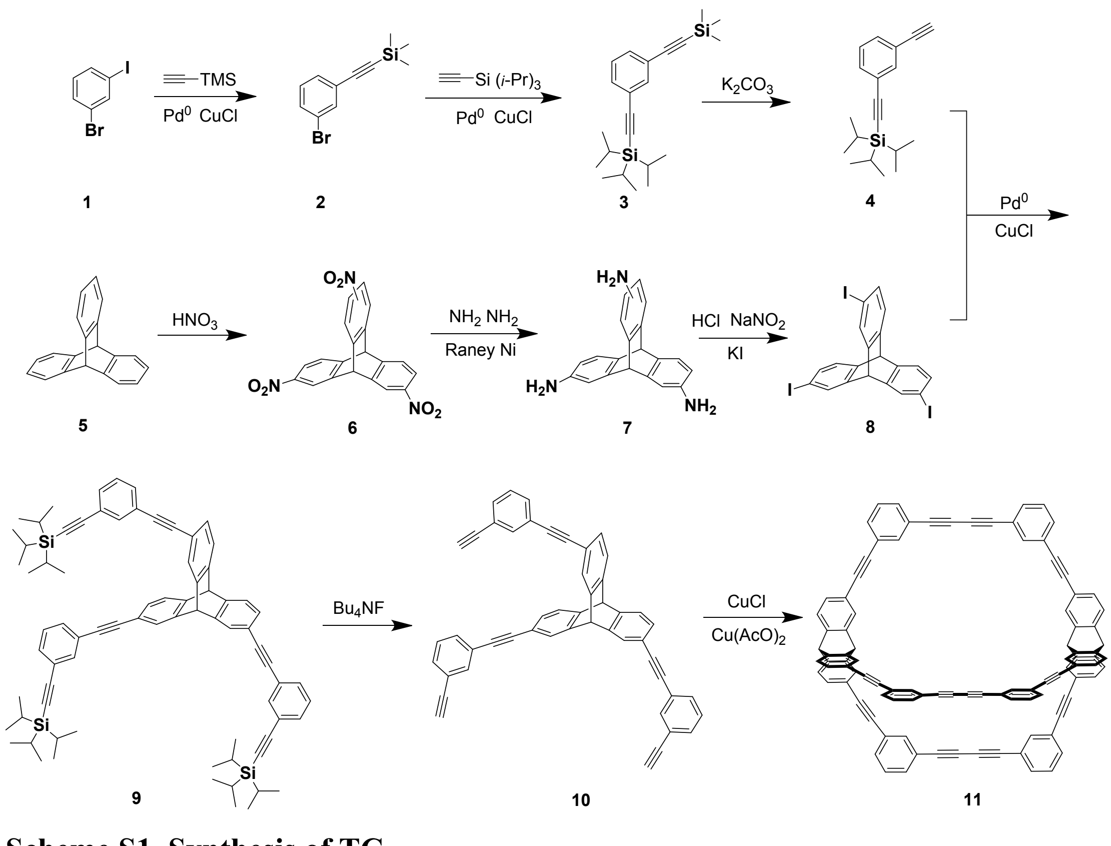

Scheme S1. Synthesis of TC.

Synthesis of **9**: The mixture of **4** (229 mg, 0.8 mmol), **8** (128 mg, 0.2 mmol), $Pd(PPh_3)_4$ (24 mg, 0.02 mmol), CuCl (3 mg, 0.03 mmol), and trimethylamine (20 mL) was stirred under argon at 75 °C for 24 h. After cooling to room temperature, the reaction mixture was concentrated in vacuo on a rotavapor and the crude was washed by 1 M HCl (3 × 30 mL). The aqueous solution was extracted with CH_2Cl_2 (3 × 30 mL), the organic

phase was combined and dried with Na_2SO_4. Compound **9** was purified by chromatography with silica gel to give a yellow solid. (118 mg, 53.39 %). ^{1}H NMR (400 MHz, $CDCl_3$, δ : ppm) 1.127 (s, 64H), 5.406 (s, 1H), 5.447 (s, 1H), 7.216 (d, J = 6.42Hz, 3H), 7.374 (d, J = 6.84Hz, 3H), 7.412 (s, 6H), 7.565 (s, 3H), 7.605 (s, 3H).

Synthesis of **10**: Compound **9** (118 mg, 0.11 mmol) was dissolved in THF (20 mL), Then a THF solution of tetrabutylammonium fluoride (0.435 mL) was added dropwise. The reaction mixture was stirred at room temperature for 20 min and evaporated. Precipitate was purified by flash chromatography to get a pale yellow solid. (65 mg, 95 %) ^{1}H NMR (400 MHz, $CDCl_3$, δ : ppm) 3.082 (s, 3H), 5.417 (s, 1H), 5.449 (s, 1H), 7.215 (dd, J = 7.62, 1.44Hz, 3H), 7.288 (t, J = 7.86Hz, 3H), 7.376 (d, J = 7.62Hz, 3H), 7.427 (d, J = 6.54Hz, 3H), 7.458 (d, J = 7.80Hz, 3H), 7.567 (s, 3H), 7.617 (s, 3H).

References

[S1] C. Zhang, C. F. Chen, *J. Org. Chem.* 2007, **72**, 9339-9341.

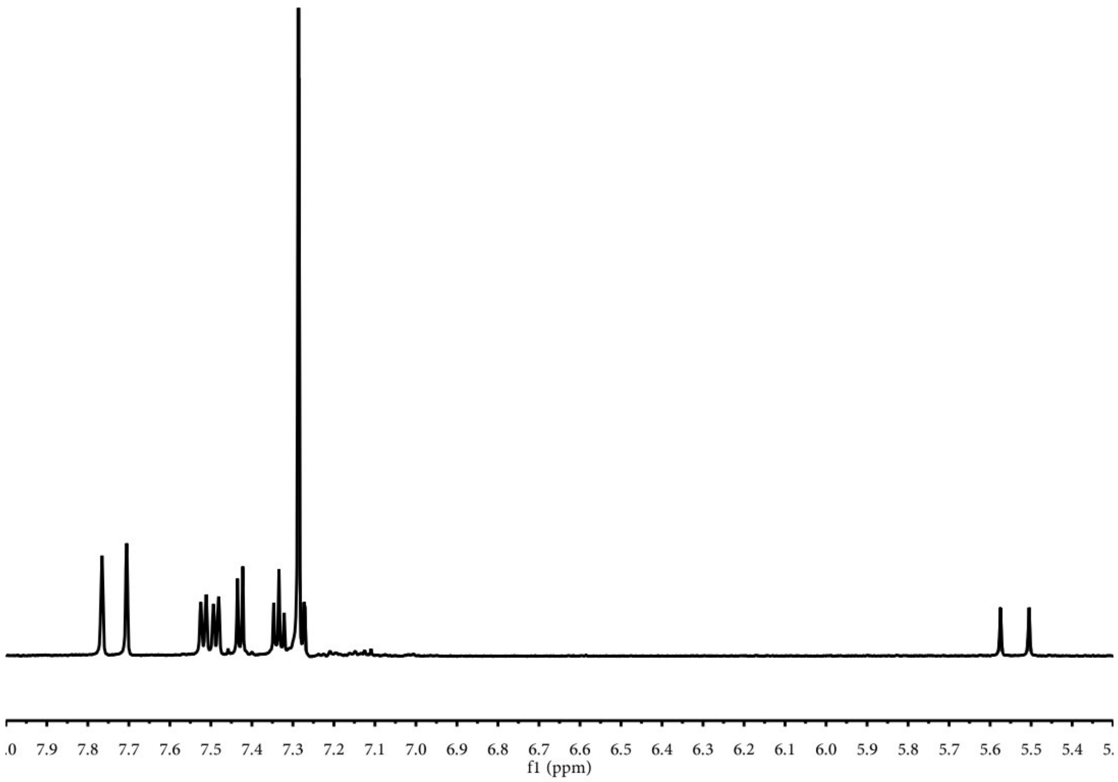

Fig. S1. ^{1}H NMR spectrum of TC.

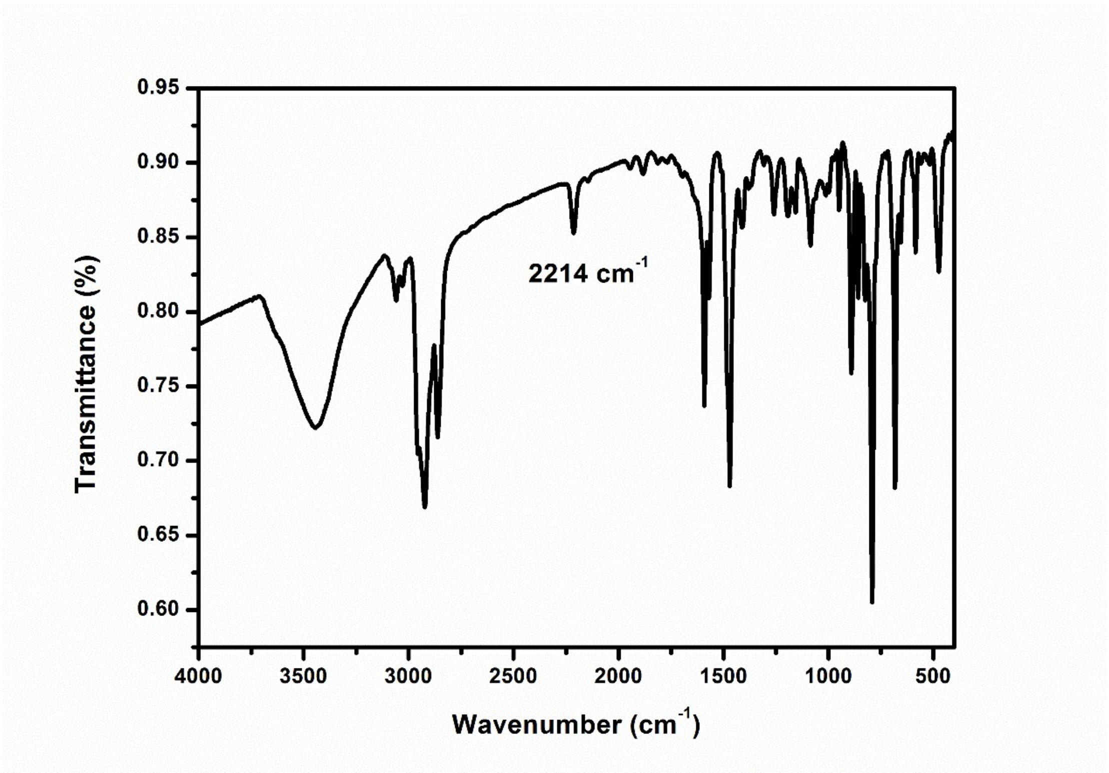

Fig. S2. FT-IR spectrum of TC.

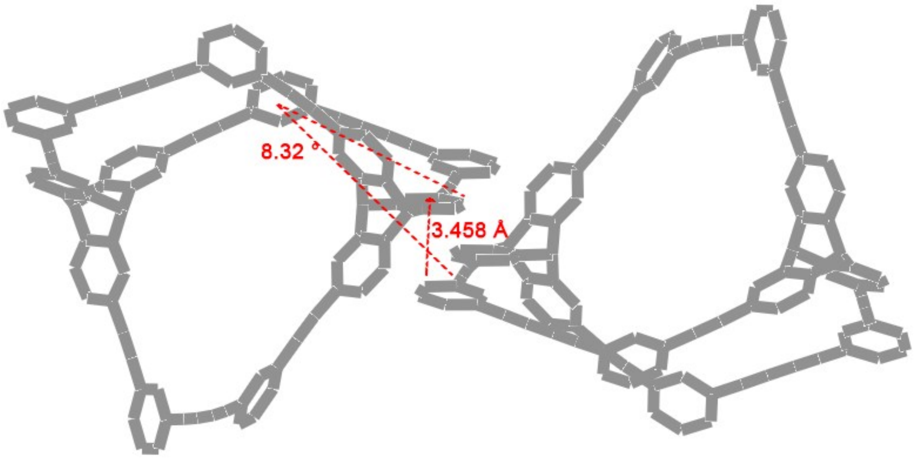

Fig. S3. View of cage molecules in the crystal packing of TC. Hydrogen atoms and solvent molecules are omitted for clarity.

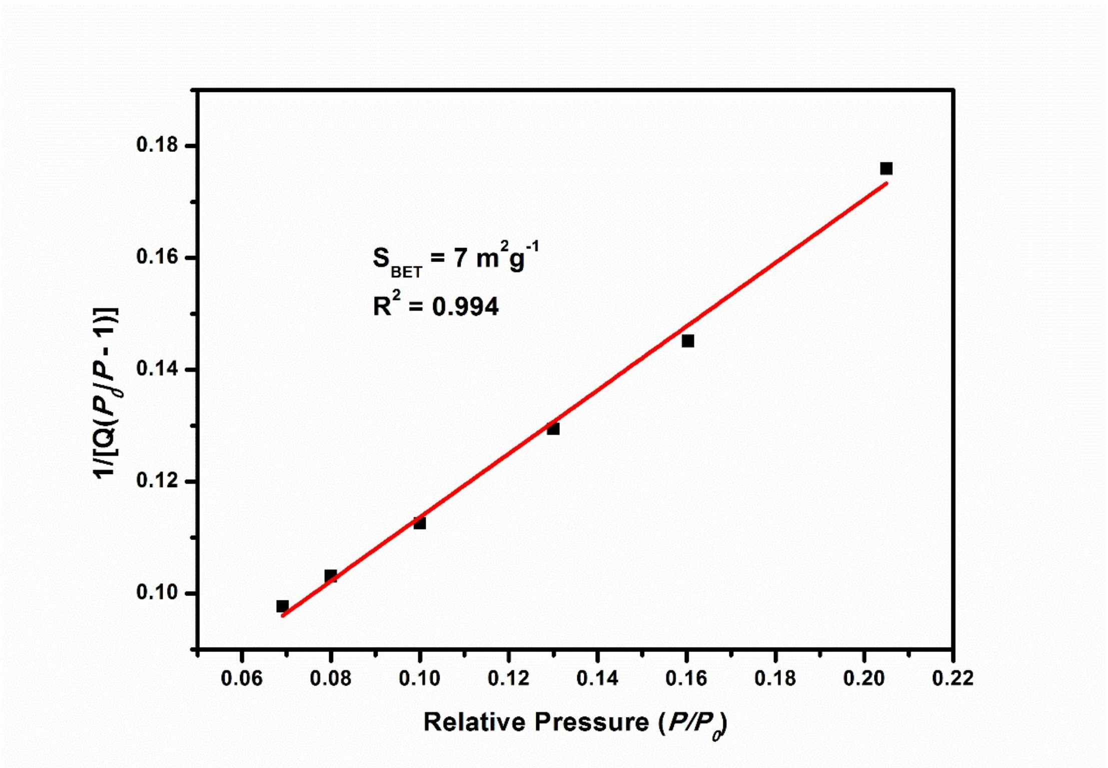

Fig. S4. BET surface area plot for TC calculated from the isotherm.

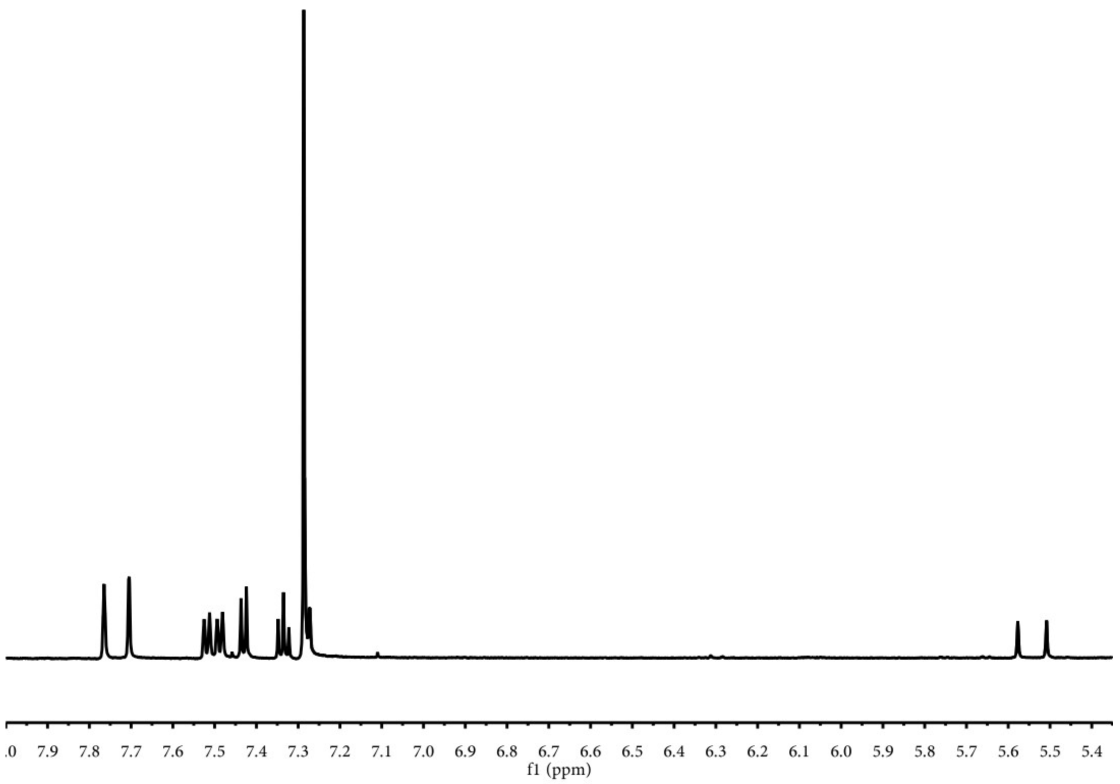

Fig. S5. ^{1}H NMR spectrum of TC-rp.

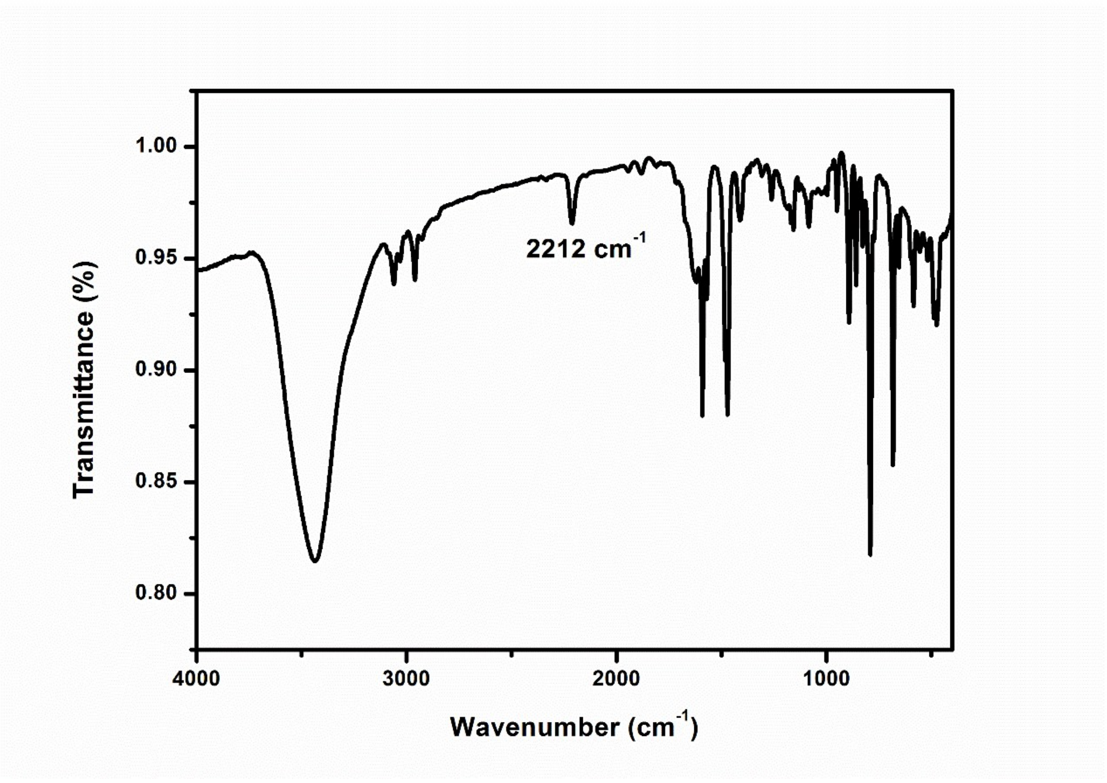

Fig. S6. FT-IR spectrum of TC-rp.

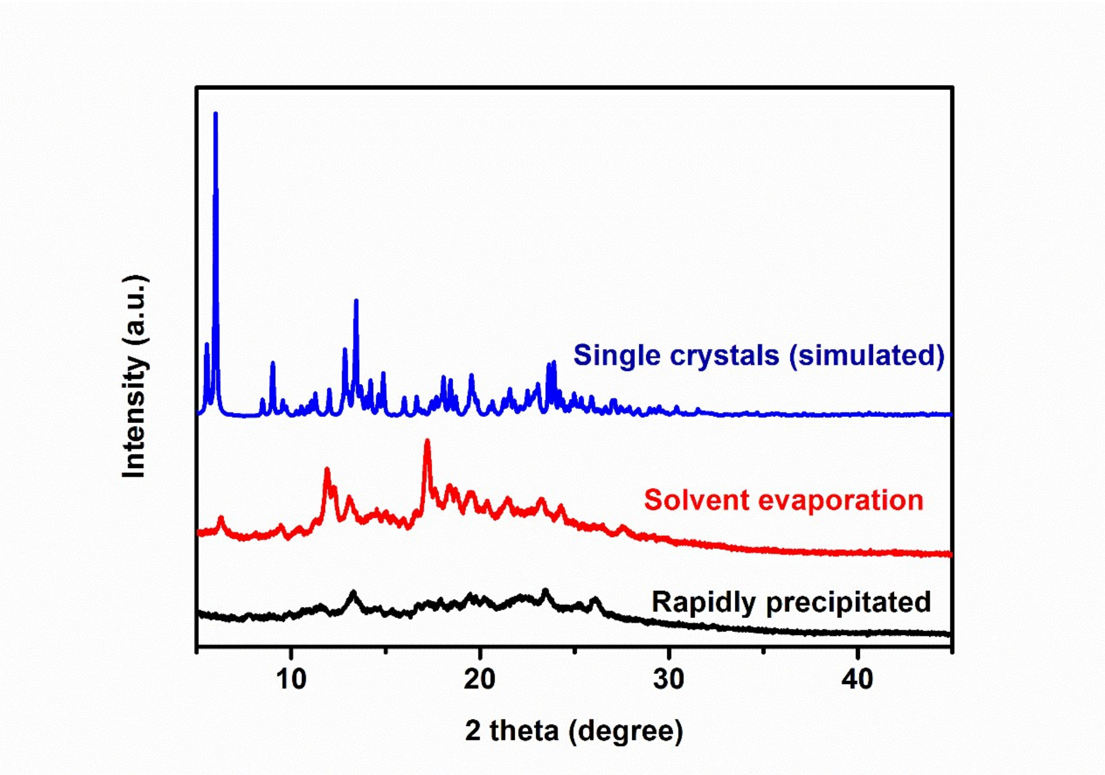

Fig. S7. PXRD patterns of simulated single crystals of TC, TC and TC-rp.

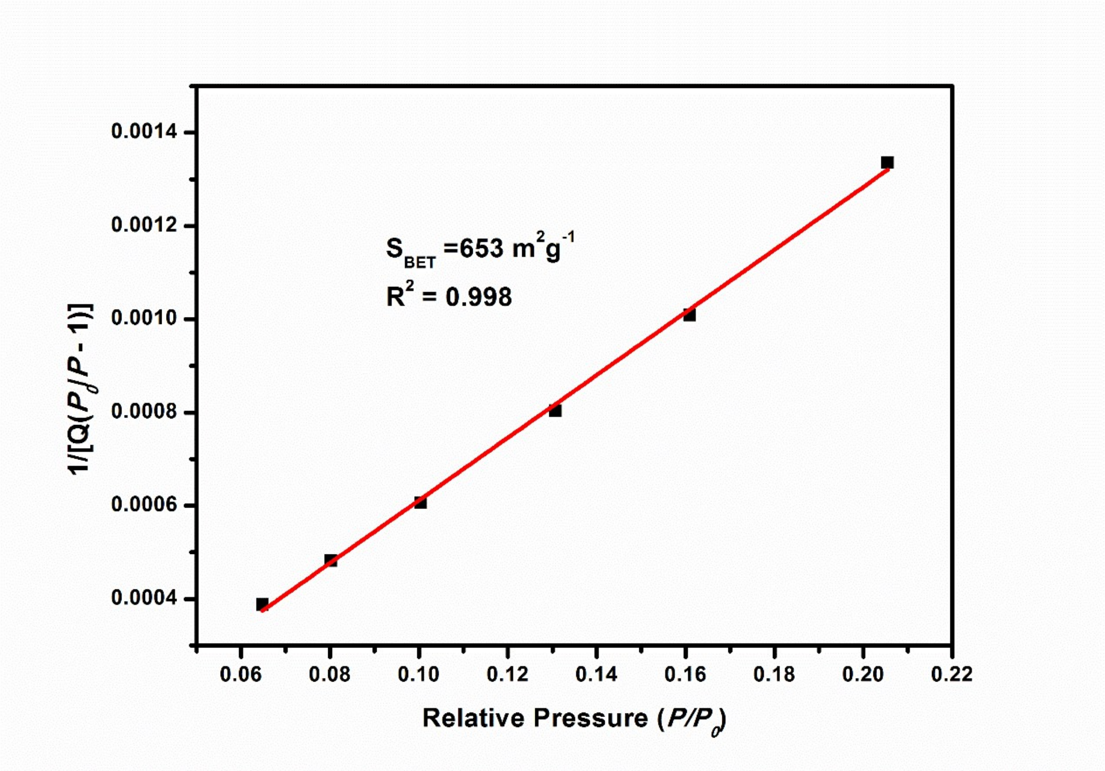

Fig. S8. BET surface area plot for TC-rp calculated from the isotherm.

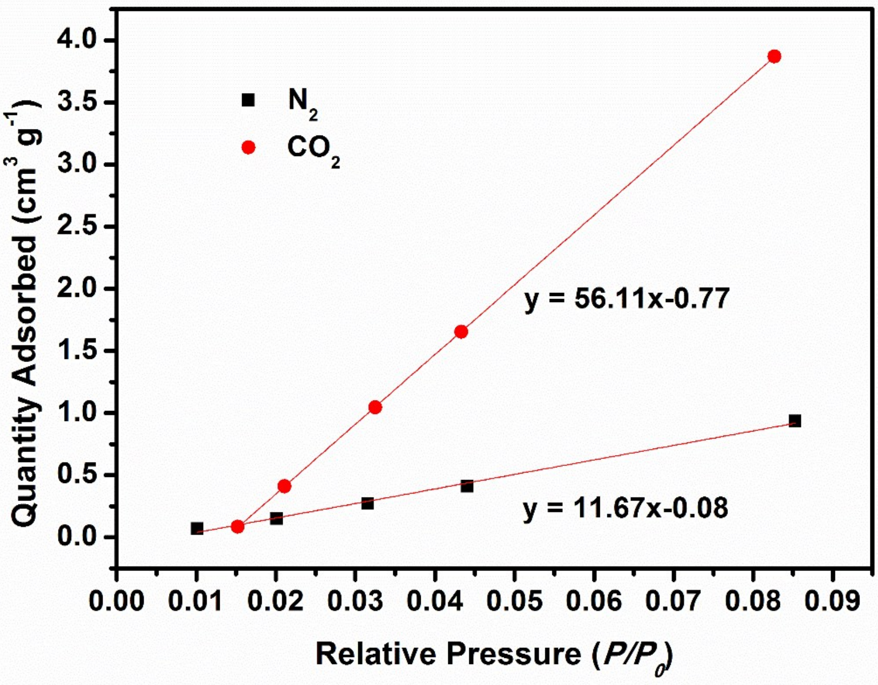

Fig. S9. Initial gas uptake slopes of TC at 273 K.

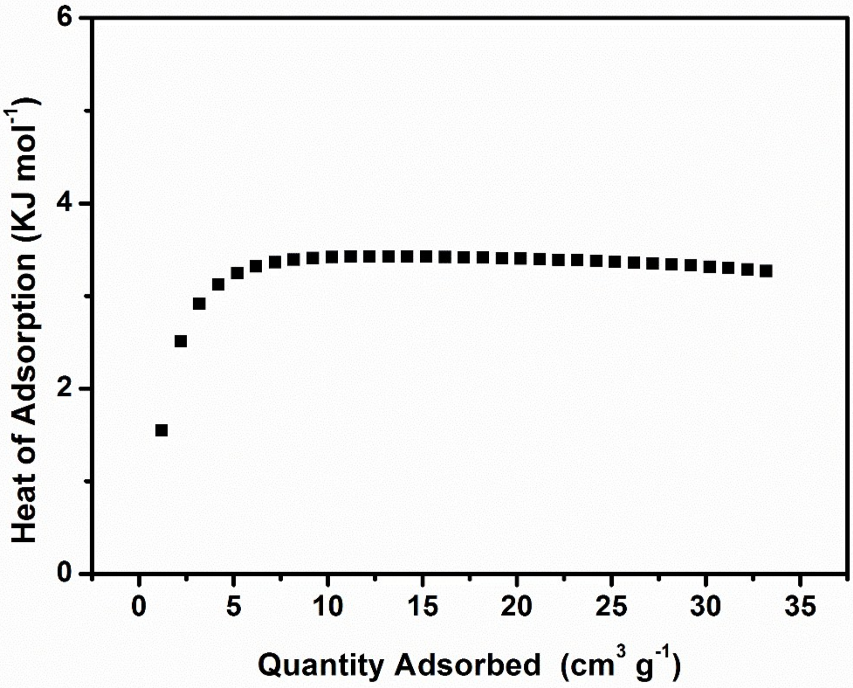

Fig. S10. Isosteric enthalpies of adsorption for CO_2 of TC.
